# Supplementary material for: Flexible Recruitments of Fundamental Muscle Synergies in the Trunk and Lower Limbs for Highly Variable Movements and Postures
Source: Sensors (Basel). 2021 Sep 15;21(18):6186. doi: 10.3390/s21186186 (PMC8472977; doi:10.3390/s21186186)
Supplement: Supplementary file 1 [file sensors-21-06186-s001.zip › sensors-1329374-supplementary.pdf]

Supplementary Information

Title: Flexible recruitments of fundamental muscle synergies in the trunk and lower limbs for highly variable movements and postures

Hiroki Saito[1,2], Hikaru Yokoyama[1], Atsushi Sasaki[1,3], Tatsuya Kato[1,3], Kimitaka Nakazawa[1*]

[1] Graduate School of Arts and Sciences, Department of Life Sciences, The University of Tokyo, 3-8-1 Komaba, Meguro, Tokyo, 153-8902, Japan.

[2] Department of Physical Therapy, Tokyo University of Technology, Ota-ku, Tokyo, 144-8535, Japan.

[3] Japan Society for the Promotion of Science, 5-3-1 Kojimachi, Chiyoda, Tokyo, 102-0083, Japan.

Correspondence to: nakazawa@idaten.c.u-tokyo.ac.jp (Kimitaka Nakazawa)

This file includes:

Figures S1, S2 and S3

Tables S1, S2 and S3

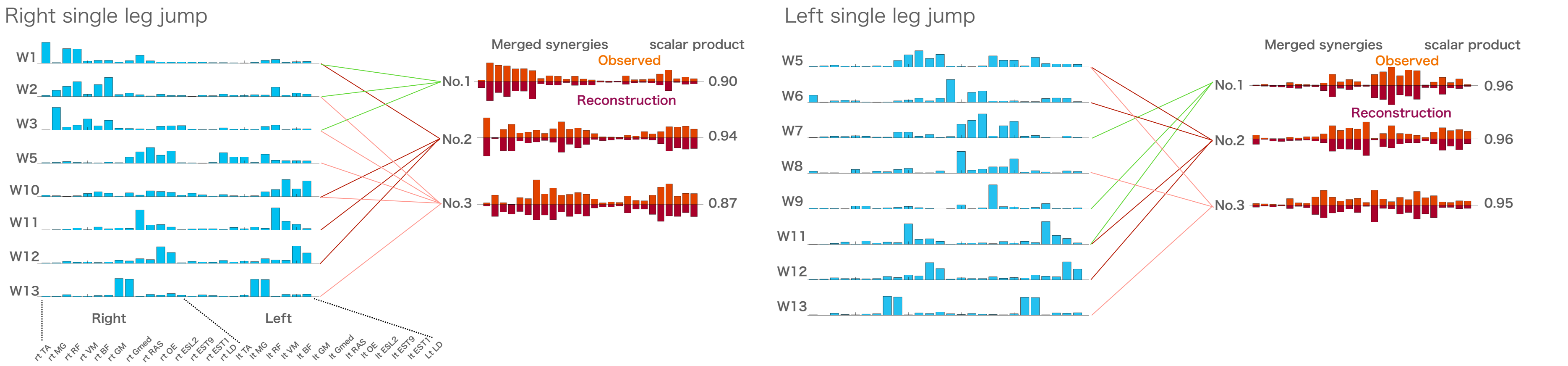

Supplementary Figure S1. Relationship between muscle synergies of all tasks and muscle synergies of other locomotion tasks. Shown are synergy cluster centroids of these task that could be explained by either a single synergy cluster or by linearly combining multiple synergy cluster centroids of all tasks (synergies in blue) matched by maximizing the scalar product to > 0.75. Observed muscle synergies extracted from the single-task EMG (orange) and their reconstructions by merging their respective W1- combinations (dark orange) are presented.

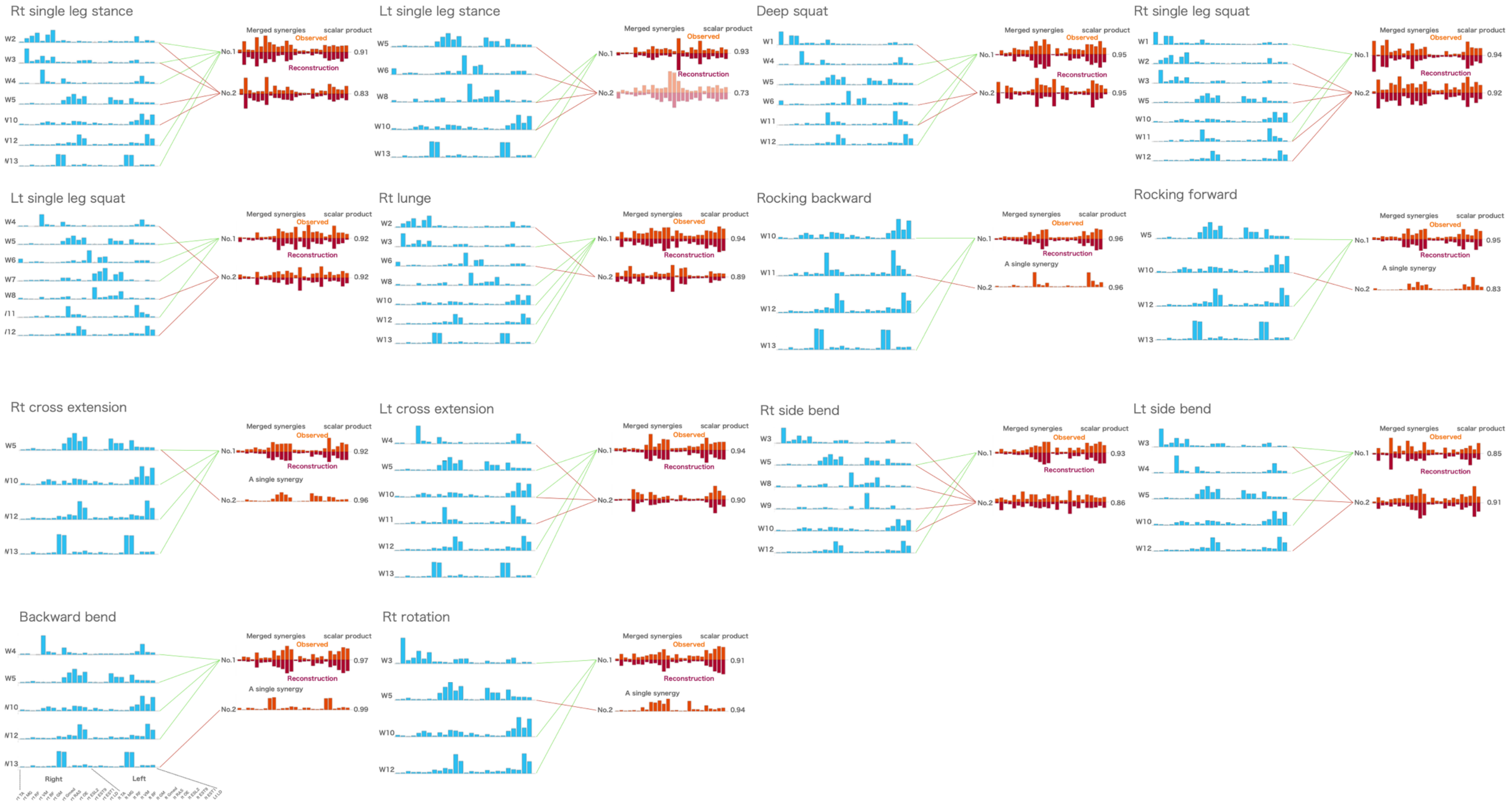
Rt single leg stance
W2
W3
W4
W5
W10
W12
W13
Merged synergies
scalar product
Observed
Reconstruction
No.1
0.91
No.2
0.83
Lt single leg stance
W5
W6
W8
W10
W13
Merged synergies
scalar product
Observed
Reconstruction
No.1
0.93
No.2
0.73
Deep squat
W1
W4
W5
W6
W11
W12
Merged synergies
scalar product
Observed
Reconstruction
No.1
0.95
No.2
0.95
Rt single leg squat
W1
W2
W3
W5
W10
W11
W12
Merged synergies
scalar product
Observed
Reconstruction
No.1
0.94
No.2
0.92
Lt single leg squat
W4
W5
W6
W7
W8
W11
W12
Merged synergies
scalar product
Observed
Reconstruction
No.1
0.92
No.2
0.92
Rt lunge
W2
W3
W6
W8
W10
W12
W13
Merged synergies
scalar product
Observed
Reconstruction
No.1
0.94
No.2
0.89
Rocking backward
W10
W11
W12
W13
Merged synergies
scalar product
Observed
Reconstruction
A single synergy
No.1
0.96
No.2
0.96
Rocking forward
W5
W10
W12
W13
Merged synergies
scalar product
Observed
Reconstruction
A single synergy
No.1
0.95
No.2
0.83
Rt cross extension
W5
W10
W12
W13
Merged synergies
scalar product
Observed
Reconstruction
A single synergy
No.1
0.92
No.2
0.96
Lt cross extension
W4
W5
W10
W11
W12
W13
Merged synergies
scalar product
Observed
Reconstruction
No.1
0.94
No.2
0.90
Rt side bend
W3
W5
W8
W9
W10
W12
Merged synergies
scalar product
Observed
Reconstruction
No.1
0.93
No.2
0.86
Lt side bend
W3
W4
W5
W10
W12
Merged synergies
scalar product
Observed
Reconstruction
No.1
0.85
No.2
0.91
Backward bend
W4
W5
W10
W12
W13
Right
Left
Merged synergies
scalar product
Observed
Reconstruction
A single synergy
No.1
0.97
No.2
0.99
Rt rotation
W3
W5
W10
W12
Merged synergies
scalar product
Observed
Reconstruction
A single synergy
No.1
0.91
No.2
0.94

Supplementary Figure S2. Relationship between muscle synergies of all tasks and muscle synergies of other stability tasks. Shown are synergy cluster centroids of these task that could be explained by either a single or linearly combining multiple synergy cluster centroids of all tasks (synergies in blue) matched by maximizing scalar product > 0.75. Observed muscle synergies extracted from the single-task EMG (orange) and their reconstructions by merging their respective W1- combinations (dark orange) were presented.

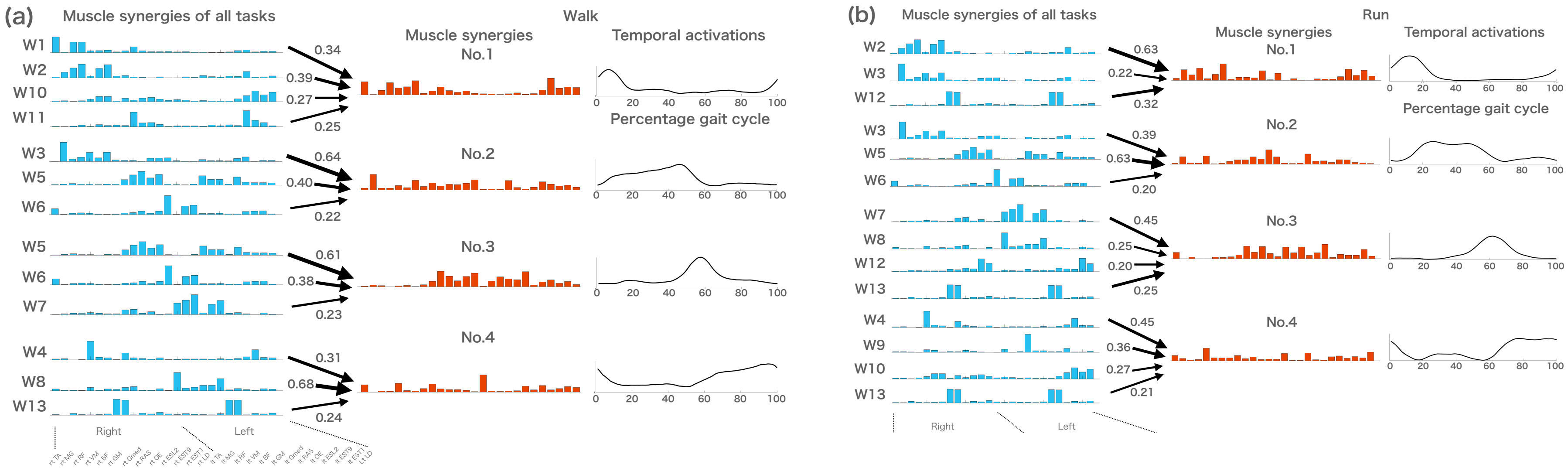

Supplementary Figure S3. Functional contributions of muscle synergies of all tasks (fundamental muscle synergies) for walking (a) and running (b). Synergy cluster centroids of all tasks were fit to a non-negative linear combination model (formula in Equation 2) to identify the merging coefficient (D in Equation 2) that best explained synergy cluster centroids of walking (a) and running (b), together with the corresponding temporal activations of each task. Here, we show that each muscle synergy cluster of all tasks (W1: 13) were flexibly recruited to meet the mechanical demands for each task. For example, in walking (a), the muscle synergy of No. 1 was recruited during the early stance phase of the right leg (i.e., initial contact, loading response to mid stance phase). Thus, W1 and W2, which contribute to the right ankle and knee stabilization and to the right knee and hip stabilization, respectively, were merged for impact deceleration and body support. The synergy cluster centroid of No. 2 was recruited during the late stance phase of the right leg (i.e., mid to terminal stance). Here, W3 with the right ankle extensor and hip stabilizer largely contributed (merging coefficient: 0.64) to the progression of the body.

Supplementary Table S1. Full descriptions of movement and postural tasks.

Categories		Task numbers	Tasks	Repetitions, duration	Conditions
Locomotion		1	Walk (1.5 m/s)	30 seconds	Walked on a treadmill (Bertec, Columbus, OH, USA) at 1.5m/s
		2	Run (2.7 m/s)	30 seconds	Run on a treadmill (Bertec, Columbus, OH, USA) at 2.7m/s
		3	Bilateral jump	8 repetitions	From a standing position, jump upward with arms freely in both sides and to maintain the same position at the instants of take-off and landing. After that, return to a standing position
		4	Rt single leg jump	8 repetitions	From standing on a right leg, jump upward with arms freely in both sides and to maintain the same position at the instants of take-off and landing
		5	Lt single leg jump	8 repetitions	From standing on a left leg, jump upward with arms freely in both sides and to maintain the same position at the instants of take-off and landing
		6	Sit to stand to sit	8 repetitions	Sit to stand to sit from a chair (40 cm height)
Stability	Static postures	7	Rt single leg stance	15 seconds	Single leg standing on a right leg
		8	Lt single leg stance	15 seconds	Single leg standing on a left leg
	Dynamic	9	Deep squat	8 repetitions	From a standing position with hands raised, squatting approximately 120 degree

	postures				of knee flexion and return
		10	Rt single leg squat	8 repetitions	From a standing position on a right leg with arms freely in both sides, squatting approximately 100 degree of knee flexion and return
		11	Lt single leg squat	8 repetitions	From a standing position on a left leg with arms freely in both sides, squatting approximately 100 degree of knee flexion and return
		12	Rt lunge	8 repetitions	From a standing position with a right leg forward, lowering a body until a left knee almost touches a floor and return
		13	Lt lunge	8 repetitions	From a standing position with a left leg forward, lowering a body until a right knee almost touches a floor and return
		14	Rocking backward	8 repetitions	In a quadruped position, transfer of the buttock backwards ("rocking") keeping low back in neutral until knees reach approximately 130 degrees of flexion and return
		15	Rocking forward	8 repetitions	In a quadruped position, transfer of the buttock forward ("rocking") keeping low back in neutral until knees reach 0 degree of hip extension and return
		16	Rt cross extension	8 repetitions	In a quadruped position, raise a right arm and a left leg straight out and return
		17	Lt cross extension	8 repetitions	In a quadruped position, raise a left arm and a right leg straight out and return

		18	Cat-and-dog	8 repetitions	In a quadruped position, round a back and drop a chin to a chest (cat) and then lift a head up and arch a back down toward a floor (dog). After that, return to a quadruped position
	Axial	19	Forward bend	8 repetitions	From a standing position, bend a trunk forward as far as possible and return
		20	Rt side bend	8 repetitions	From a standing position, bend a trunk to a right side as far as possible and return
		21	Lt side bend	8 repetitions	From a standing position, bend a trunk to a left side as far as possible and return
		22	Backward bend	8 repetitions	From a standing position with arms raised, bend a trunk backward as far as possible and return
		23	Rt rotation	8 repetitions	From a standing position, rotate a trunk to a right side as far as possible and return
		24	Lt rotation	8 repetitions	From a standing position, rotate a trunk to a left side as far as possible and return

Each participants performed 24 locomotion and stability tasks. The order of tasks was randomly assigned. For each repetition, the starting point was initiated with the verbal cue 'go' for 1 s after the resting posture. A movement period of approximately 6 s followed by a resting period of 1 s was given to complete the tasks. After the examiner visually ensured that the task was completed along with the resting period, the end of the period was indicated by a second verbal cue 'end'. For the right and left single-leg stance tasks, the starting point was defined as the point at which participants performed a single-leg stance and became stable, and the end of the movement was 15 s after the starting point.

Supplementary Table S2: Summary of results for the mean number of synergies, mean VAF of each task in the subjects, and the degree of similarity within each synergy cluster of each task across subjects.

Locomotion tasks							
		Walk	Run	BilJP	Rt SJP	Lt SJP	STS
No synergies of each task		4.70 (± 1.49)	4.40 (± 0.84)	3.60 (± 0.97)	3.60 (± 1.07)	3.90 (± 1.60)	2.30 (± 0.67)
VAF mean of each task		0.96 (± 0.01)	0.96 (± 0.01)	0.97 (± 0.01)	0.97 (± 0.01)	0.97 (± 0.01)	0.97 (± 0.01)
Degree of similarity within each synergy cluster of each task	W1	0.73 (± 0.10)	0.85 (± 0.04)	0.68 (± 0.20)	0.69 (± 0.16)	0.88 (± 0.04)	0.70 (± 0.17)
	W2	0.57 (± 0.21)	0.64 (± 0.20)	0.62 (± 0.16)	0.79 (± 0.06)	0.70 (± 0.14)	0.72 (± 0.20)
	W3	0.75 (± 0.10)	0.81 (± 0.07)	0.86 (± 0.07)	0.71 (± 0.14)	0.53 (± 0.18)	//
	W4	0.65 (± 0.21)	0.59 (± 0.22)	0.84 (± 0.06)	//	//	//

Stability tasks (static and dynamic postures)													
		Rt SLS	Lt SLS	DS	Rt SS	Lt SS	Rt LG	Lt LG	RB	RF	Rt CE	Lt CE	CD
No synergies of each task		1.00 (± 0.00)	1.30 (± 0.67)	2.10 (± 0.32)	2.20 (± 0.42)	2.40 (± 0.97)	2.90 (± 1.10)	2.80 (± 1.55)	1.50 (± 0.53)	1.60 (± 0.70)	2.10 (± 0.57)	2.20 (± 0.42)	1.90 (± 0.88)
VAF mean of each task		0.97 (± 0.01)	0.97 (± 0.01)	0.97 (± 0.01)	0.97 (± 0.01)	0.97 (± 0.01)	0.97 (± 0.01)	0.97 (± 0.01)	0.97 (± 0.01)	0.97 (± 0.01)	0.97 (± 0.01)	0.97 (± 0.01)	0.97 (± 0.01)
Degree of similarity within each synergy cluster of each task	W1	0.68 (± 0.11)	0.62 (± 0.18)	0.80 (± 0.08)	0.81 (± 0.06)	0.64 (± 0.21)	0.63 (± 0.18)	0.74 (± 0.09)	0.80 (± 0.10)	0.70 (± 0.13)	0.76 (± 0.08)	0.76 (± 0.11)	0.80 (± 0.07)
	W2	0.80 (± 0.05)	0.57 (± 0.20)	0.80 (± 0.12)	0.61 (± 0.18)	0.72 (± 0.11)	0.65 (± 0.19)	0.48 (± 0.24)	0.86 (± 0.08)	0.80 (± 0.09)	0.82 (± 0.08)	0.77 (± 0.10)	0.74 (± 0.12)
	W3	//	//	//	//	//	//	//	//	//	//	//	0.92 (± 0.06)
	W4	//	//	//	//	//	//	//	//	//	//	//	//

		Stability tasks (Axial)					
		FB	Rt SB	Lt SB	BB	Rt RT	Lt RT
No synergies of each task		2.30 (± 0.95)	2.20 (± 1.81)	2.00 (± 1.49)	1.90 (± 0.57)	2.00 (± 0.67)	2.00 (± 0.94)
VAF mean of each task		0.97 (± 0.01)	0.97 (± 0.01)	0.97 (± 0.01)	0.97 (± 0.01)	0.97 (± 0.01)	0.97 (± 0.01)
Degree of similarity within each synergy cluster of each task	W1	0.64 (± 0.18)	0.76 (± 0.11)	0.50 (± 0.18)	0.77 (± 0.11)	0.69 (± 0.13)	0.56 (± 0.23)
	W2	0.66 (± 0.18)	0.37 (± 0.19)	0.72 (± 0.09)	0.85 (± 0.12)	0.66 (± 0.16)	0.81 (± 0.08)
	W3	//	//	//	//	//	//
	W4	//	//	//	//	//	//

The mean (SD) number of muscle synergies extracted from single-task matrices, the mean (SD) VAF of each task in each participant and the mean (SD) of the degree of similarity within each cluster of each task by averaging the values of all the pairwise scalar products between synergies that compose each cluster. Bil, bilateral; Rt, right; Lt, left; JP, jump; SJP, single-leg jump; STS: sit-to-stand-to-sit; SLS, single-leg stance; DS, deep squat; SS, single-leg squat; LG, lunge; RB, rocking backward; RF, rocking forward; CE, cross extension; CD, cat-and-dog; FB, forward bend; SB, side bend; BB, backward bend; RT, rotation.

Supplementary Table S3: Recruitment coefficients of 13 synergy clusters of all tasks for each task execution.

		Walk	Run	BilJP	Rt SJP	Lt SJP	STS	Rt SLS	Lt SLS	DS	Rt SS	Lt SS	Rt LG	Lt LG	RB	RF	Rt CE	Lt CE	CD	FB	Rt SB	Lt SB	BB	Rt RT	Lt RT
Right patterns	C1	0.92	//	0.97	**0.94**	//	0.96	//	//	0.98	0.98	//	//	0.97	//	//	//	//	//	//	//	//	//	//	//
	C2	0.92	1.00	0.95	**0.98**	//	//	**0.99**	//	//	0.94	//	0.91	0.87	//	//	//	//	//	//	//	//	//	//	//
	C3	0.97	**0.85**	**0.92**	**0.93**	//	//	**0.99**	//	//	0.98	//	0.97	//	//	//	//	//	//	0.94	0.97	**0.94**	//	0.97	//
	C4	0.87	0.87	//	//	//	//	0.99	//	0.97	//	0.88	//	//	//	//	//	0.99	//	0.98	//	0.99	0.93	//	0.97
	C5	**0.98**	0.91	0.99	0.91	**0.96**	0.99	1.00	0.99	0.93	0.95	0.91	//	0.99	//	0.94	**0.99**	0.99	0.98	0.96	**0.99**	**0.98**	0.99	0.99	//
Left patterns	C6	**0.90**	0.88	0.99	//	0.99	0.98	//	0.98	0.98	//	0.96	0.98	0.97	//	//	//	//	//	//	//	//	//	//	//
	C7	0.87	0.98	0.94	//	0.97	//	//	//	//	//	0.96	//	0.90	//	//	//	//	//	//	//	//	//	//	//
	C8	0.97	0.92	0.85	//	0.96	//	//	**0.99**	//	//	0.99	0.95	0.98	//	//	//	//	//	//	0.95	//	//	//	//
	C9	//	0.76	//	//	0.87	//	//	//	//	//	//	//	//	//	//	//	//	//	0.96	0.94	//	//	//	//
	C10	0.90	0.96	0.92	**0.95**	//	//	0.99	**0.98**	//	0.94	//	0.96	//	0.99	0.93	0.99	**0.99**	0.97	0.95	**0.98**	0.99	0.98	0.99	0.99
Bilateral patterns	C11	0.80	0.77	0.90	0.92	**0.88**	**0.97**	//	//	**0.97**	**0.98**	0.97	//	0.93	0.98	//	//	0.96	0.95	0.90	//	//	//	//	//
	C12	//	**0.94**	0.97	0.91	0.91	0.99	0.99	//	0.96	0.97	0.93	0.99	0.99	1.00	0.99	0.98	0.98	0.99	0.99	1.00	1.00	0.99	0.98	**0.99**
	C13	0.89	0.93	**0.94**	0.98	0.98	//	1.00	1.00	//	//	//	0.97	//	0.99	0.98	0.97	0.98	**0.98**	0.90	//	//	1.00	//	//

Recruitment coefficients (RC) of each 13-synergy cluster centroid of all tasks for each task execution. To quantitatively assess whether synergy cluster centroids of all tasks in independent and/or merging states were recruited for each task execution, RC were calculated as the similarity (scalar product) between the

temporal activation of a synergy cluster centroid of all tasks (C1:13) and the corresponding temporal activations of each task. Of note, similarities were calculated only for synergy cluster centroids of all tasks that contributed to each task execution (i.e., Figure 2, 3, S2 and S3). If a synergy cluster centroid of all tasks contributed to more than one synergy cluster centroid of each task, we assessed whether the temporal activation of a synergy cluster centroid of all tasks was reconstructed by a linear combination of the corresponding temporal activations of each task. Thus, temporal activations of each task were fitted to a non-negative linear combination model to identify the combination coefficient that best explained the temporal activation of all tasks using a similar formula as in Equation 2. Then, we calculated the similarity between the reconstructed temporal activation of each task and the corresponding temporal activation of all tasks (bold). Bil, bilateral; Rt, right; Lt left; JP, jump; SJP, single-leg jump; STS, sit-to-stand-to-sit; SLS, single-leg stance; DS, deep squat; SS, single-leg squat; LG, lunge; RB, rocking backward; RF, rocking forward; CE, cross extension; CD, cat-and-dog; FB, forward bend; SB, side bend; BB, backward bend; RT, rotation.
